# Structural basis of pyrimidine-pyrimidone (6–4) photoproduct recognition by UV-DDB in the nucleosome

**DOI:** 10.1038/srep16330

**Published:** 2015-11-17

**Authors:** Akihisa Osakabe, Hiroaki Tachiwana, Wataru Kagawa, Naoki Horikoshi, Syota Matsumoto, Mayu Hasegawa, Naoyuki Matsumoto, Tatsuya Toga, Junpei Yamamoto, Fumio Hanaoka, Nicolas H. Thomä, Kaoru Sugasawa, Shigenori Iwai, Hitoshi Kurumizaka

**Affiliations:** 1 Laboratory of Structural Biology, Graduate School of Advanced Science and Engineering, Waseda University, 2-2 Wakamatsu-cho, Shinjuku-ku, Tokyo 162-8480, Japan; 2 Department of Interdisciplinary Science and Engineering, Program in Chemistry and Life Science, School of Science and Engineering, Meisei University, 2-1-1 Hodokubo, Hino-shi, Tokyo 191-8506, Japan; 3 Biosignal Research Center, Organization of Advanced Science and Technology, Kobe University, 1-1 Rokkodai-cho, Nada-ku, Kobe-shi, Hyogo 657-8501, Japan; 4 Graduate School of Engineering Science, Osaka University, 1-3 Machikaneyama, Toyonaka-shi, Osaka 560-8531, Japan; 5 Faculty of Science, Gakushuin University, 1-5-1 Mejiro, Toshima-ku, Tokyo 171-8588, Japan; 6 Friedrich Miescher Institute for Biomedical Research, Maulbeerstrasse 66, CH 4058 Basel, Switzerland

## Abstract

UV-DDB, an initiation factor for the nucleotide excision repair pathway, recognizes 6–4PP lesions through a base flipping mechanism. As genomic DNA is almost entirely accommodated within nucleosomes, the flipping of the 6–4PP bases is supposed to be extremely difficult if the lesion occurs in a nucleosome, especially on the strand directly contacting the histone surface. Here we report that UV-DDB binds efficiently to nucleosomal 6–4PPs that are rotationally positioned on the solvent accessible or occluded surface. We determined the crystal structures of nucleosomes containing 6–4PPs in these rotational positions, and found that the 6–4PP DNA regions were flexibly disordered, especially in the strand exposed to the solvent. This characteristic of 6–4PP may facilitate UV-DDB binding to the damaged nucleosome. We present the first atomic-resolution pictures of the detrimental DNA cross-links of neighboring pyrimidine bases within the nucleosome, and provide the mechanistic framework for lesion recognition by UV-DDB in chromatin.

Pyrimidine-pyrimidone (6–4) photoproducts (6–4PPs) and cyclobutane pyrimidine dimers (CPDs) are induced by ultraviolet (UV) light in genomic DNA^1^,^2^. In mammals, these UV-induced DNA photolesions are repaired by the nucleotide excision repair (NER) pathway^1^,^2^. A damage surveillance protein complex, UV-damaged DNA-binding (UV-DDB) protein, plays a pivotal role in the initial stage of the NER process^3^, and is implicated in the human autosomal recessive disorder xeroderma pigmentosum. UV-DDB is composed of the DDB1 and DDB2 subunits^4^,^5^, and is capable of binding 6–4PP more tightly than CPD^3^,^6^,^7^,^8^,^9^. The crystal structure of UV-DDB complexed with 6–4PP/CPD DNA revealed that the photodimer is flipped from the helical DNA axis^10^,^11^.

In eukaryotes, genomic DNA is packaged in chromatin, with nucleosomes as the repeating unit. A nucleosome is composed of 147 base-pairs of DNA^12^ and a histone octamer, including two copies each of H2A, H2B, H3, and H4^13^. Chromatin functions as the template for most reactions involved in DNA metabolism, including DNA replication, transcription, and repair. Key DNA repair processes, including NER, must thus operate in the context of chromatin. However, DNA repair may be impeded in the context of the nucleosome, which generally restricts the accessibility of the DNA-binding proteins, including UV-DDB. Therefore, it is currently unclear how UV-DDB recognizes 6–4PP lesions in the context of chromatin.

In the present study, we reconstituted two types of nucleosomes containing 6–4PPs, rotationally positioned to face either the solvent or the histones. We found that UV-DDB efficiently binds to the nucleosomal 6–4PP in both rotational positions. We then determined the crystal structures of these nucleosomes, which revealed that the 6–4PP DNA regions are flexibly disordered especially in the strand exposed to the solvent, regardless of whether the 6–4PP bases are present in the strand. We also found that UV-DDB exhibited higher binding activity to nucleosomes containing the apyrimidinic (AP) site on the strand exposed to the solvent. The DNA backbone of the strand containing the AP site is generally more flexible. Therefore, UV-DDB may first recognize the flexible DNA backbone at the 6–4PP site in the nucleosome, and form a stable complex with the flipped damaged bases, probably with the aid of nucleosome remodelers and/or histone removal.

## Results

### UV-DDB efficiently binds to the 6–4PPs in nucleosomes

**Figure 1:**
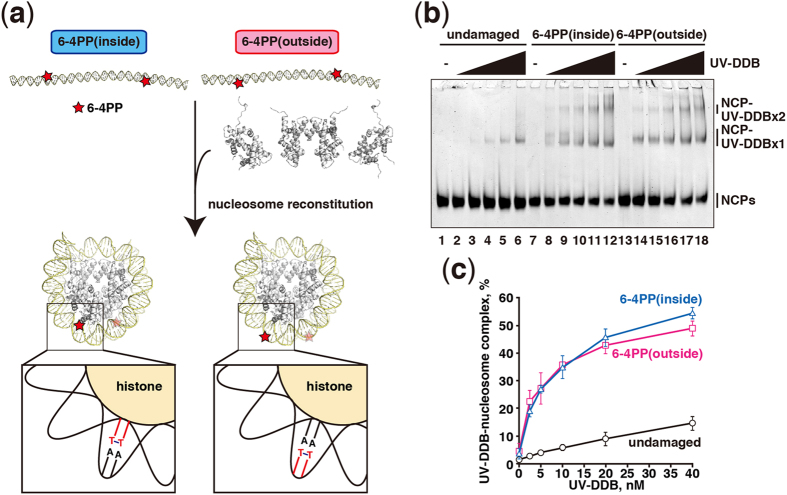
Nucleosomal 6–4PP DNA binding of UV-DDB. (**a**) Schematic representations of reconstituted nucleosomes containing 6–4PP(inside) and 6–4PP(outside). The affected T-T bases are indicated in red. (**b**) Gel electrophoretic mobility shift assay for nucleosome binding by UV-DDB. Nucleosome core particles (NCP; 5 nM) containing undamaged DNA (lanes 1–6), 6–4PP(inside) (lanes 7–12), or 6–4PP(outside) (lanes 13–18) were incubated with UV-DDB. The UV-DDB concentrations are 0 nM (lanes 1, 7, and 13), 2.5 nM (lanes 2, 8, and 14), 5 nM (lanes 3, 9, and 15), 10 nM (lanes 4, 10, and 16), 20 nM (lanes 5, 11, and 17), and 40 nM (lanes 6, 12, and 18). (**c**) Graphic representation of the experiments shown in panel (**b**). Standard deviation values are shown (n = 3).

To study the UV-DDB binding to the nucleosomal 6–4PPs, we reconstituted the nucleosome core particles with a palindromic DNA sequence, in which two 6–4PPs were introduced at symmetric sites (Supplementary Fig. S1). These symmetric positions of the 6–4PPs within a nucleosome facilitated the determination of the crystal structures of nucleosomal 6–4PPs (see below), although they may not occur in such close proximity *in vivo*. To detect the nucleosome-UV-DDB complex, nucleosomes were reconstituted with histones H2A, H3.1, H4, and the H2B T122C mutant, in which the Thr122 residue of H2B was replaced by Cys for fluorescent labeling by Alexa488. We then performed the nucleosome binding assay with the purified UV-DDB and purified Alexa488-labeled nucleosomes (Supplementary Figs S2 and S3). To do so, the 6–4PP(outside) and 6–4PP(inside) nucleosomes, in which the 6–4PPs were rotationally positioned in a DNA strand exposed toward the solvent and contacting the histone surface, respectively, were prepared (Fig. 1a). The 6–4PP positions within the nucleosomes were confirmed by X-ray crystallography (see below).

The nucleosome binding assay was conducted in the presence of a 19-fold molar excess amount of unlabeled nucleosomes without 6–4PP lesions. Under these conditions, UV-DDB binding to the undamaged nucleosome was only weakly detected, while UV-DDB bound tightly to the 6–4PP(outside) nucleosome (Fig. 1b,c). We detected two bands with the 6–4PP(outside) nucleosome in the presence of UV-DDB (Fig. 1b). The lower and upper bands represent the nucleosomes complexed with one UV-DDB and two UV-DDBs, respectively. Unexpectedly, UV-DDB also bound to the 6–4PP(inside) nucleosome with comparable efficiency to the 6–4PP(outside) nucleosome (Fig. 1b,c).

A third species, just above the major UV-DDB-nucleosome complex band (the lower band), was specifically observed in the UV-DDB binding experiments with the 6–4PP(inside) nucleosome (Fig. 1b, lanes 8–10). This suggests that UV-DDB has two binding modes to the 6–4PP(inside) nucleosome, although the underlying mechanisms are not currently understood.

### Crystal structures of the 6–4PP nucleosomes

**Figure 2:**
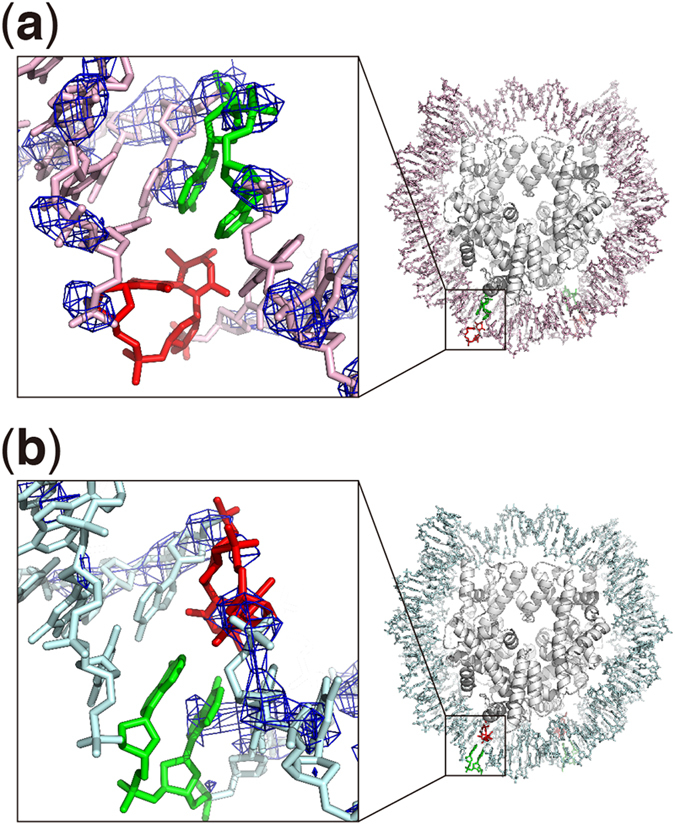
Crystal structures of the nucleosomes containing 6–4PP(outside) and 6–4PP(inside). (**a**) Structure of the nucleosome containing 6–4PP(outside). (**b**) Structure of the nucleosome containing 6–4PP(inside). The left panels show close-up views of 6–4PP (red) and its complementary bases (green) in the nucleosomes. The *2mFo-DFc* maps (contoured at 1.5 σ and colored blue) surround stick representations of the DNA around the 6–4PP residues.

We then determined the crystal structures of the nucleosomes containing the 6–4PP(outside) or 6–4PP(inside) lesions (Fig. 2, Supplementary Table S1). For the nucleosome crystallization, we used 6–4PP(outside) and 6–4PP(inside) DNAs containing sequences identical to those used in the nucleosome binding assays (Supplementary Fig. S1). Our design ensured that, in the crystals, one of the two 6–4PP DNA regions was exposed to the solvent without a crystal packing contact. As expected, in both the 6–4PP(outside) and 6–4PP(inside) nucleosome structures, one 6–4PP site is completely exposed to the solvent, and no direct contact is observed with other nucleosome molecules in the crystal lattice (Supplementary Fig. S4).

Two types of solution structures, one with a sharp kink^14^,^15^ and the other without a large distortion^16^,^17^, have been reported for 6–4PP-containing DNA. In the present nucleosome structures, the DNA regions around the 6–4PP sites are not kinked within the nucleosomal DNA (Fig. 2). Actually, the 6–4PP DNA model with the same kinking angle as that in a previously published 6–4PP DNA structure^14^ did not fit well with the nucleosomal 6–4PP DNA structure (Supplementary Fig. S5). However, in both the 6–4PP(outside) and 6–4PP(inside) nucleosomes, the electron densities around the 6–4PPs are quite ambiguous in both strands, indicating that the DNA region containing the 6–4PP is flexible in the nucleosomes (Fig. 2a,b). Especially, in the 6–4PP(outside) nucleosome, the affected thymine dimer nucleotides are entirely disordered (Fig. 2a). The 6–4PP thus may become flipped-out more easily, if it exists on the strand exposed to the solvent. Scrima *et al.* reported that UV-DDB flips 6–4PPs out of the DNA double helix, and directly interacts with both strands of the damaged site^10^. The superimposition of the present structure with the UV-DDB (DDB2)-6–4PP DNA complex illustrates that DDB2 is capable of recognizing 6–4PP within the nucleosome, if it is located on the strand exposed to the solvent (Supplementary Fig. S6).

In the 6–4PP(inside) nucleosome structure, the electron densities for the 6–4PP bases are interpretable, and the 6–4PP bases are not flipped out of the DNA double helix (Fig. 2b). However, we found that the electron densities for the backbone atoms of the strand complementary to the 6–4PP bases are extremely weak, as compared to the electron densities outside the lesion (Fig. 2b). These findings suggest that, in the nucleosome, the DNA backbone at the 6–4PP sites is disordered in the strand exposed to the solvent, regardless of the existence of 6–4PP bases in the flexible strand.

### UV-DDB preferentially binds to damaged nucleosomes containing a flexible strand

**Figure 3:**
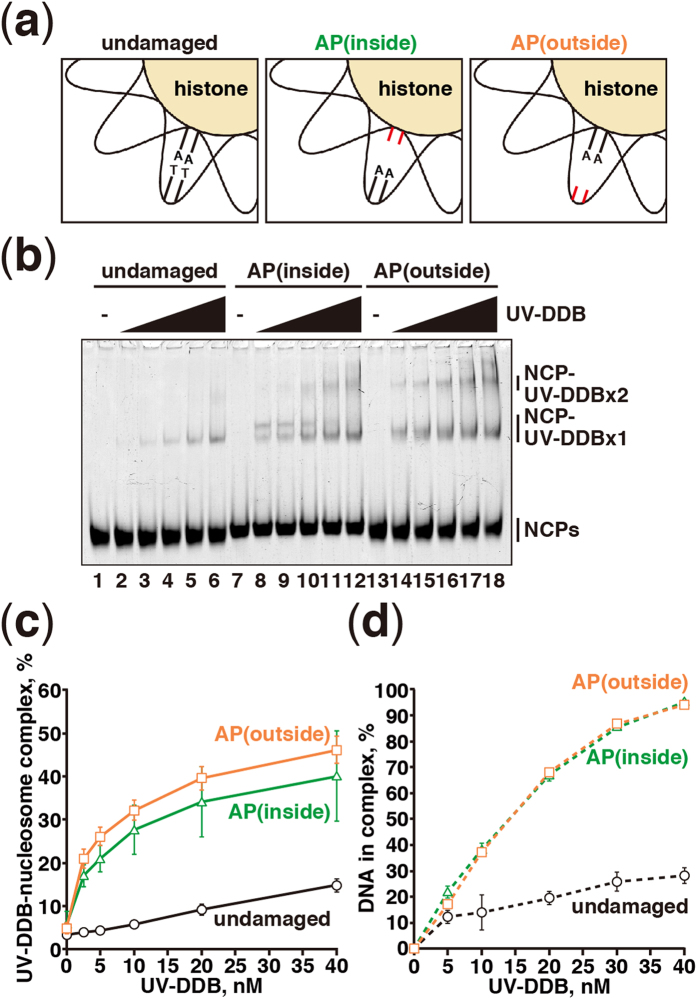
Nucleosomal apyrimidinic DNA binding of UV-DDB. (**a**) Schematic representations of reconstituted nucleosomes containing undamaged DNA, AP(inside), and AP(outside). (**b**) Gel electrophoretic mobility shift assay for nucleosome binding of UV-DDB. Nucleosome core particles (NCP; 5 nM) containing undamaged DNA (lanes 1–6), AP(inside) (lanes 7–12), or AP(outside) (lanes 13–18) were incubated with UV-DDB. The UV-DDB concentrations are 0 nM (lanes 1, 7, and 13), 2.5 nM (lanes 2, 8, and 14), 5 nM (lanes 3, 9, and 15), 10 nM (lanes 4, 10, and 16), 20 nM (lanes 5, 11, and 17), and 40 nM (lanes 6, 12, and 18). (**c**) Graphic representation of the experiments shown in panel (**b**). Standard deviation values are shown (n = 3). (**d**) Graphic representation of naked DNA binding of UV-DDB. Standard deviation values are shown (n = 3).

To test whether the UV-DDB binding occurs on the region containing a flexible DNA strand, we reconstituted the AP(outside) and AP(inside) nucleosomes. These represented model nucleosomes containing a flexible strand, and contained two missing consecutive thymine bases in a strand exposed to the solvent or contacting the histone surface, respectively (Fig. 3a, Supplementary Fig. S2c). Interestingly, we found that UV-DDB robustly bound to the AP(outside) nucleosome, although the bases were missing on the strand exposed to the solvent (Fig. 3b,c). The UV-DDB binding to the AP(inside) nucleosome was slightly less efficient, as compared to the binding to the AP(outside) nucleosome (Fig. 3b,c). However, we confirmed that UV-DDB bound to the naked AP(outside) and AP(inside) DNAs with exactly equal efficiency (Fig. 3d).

**Figure 4:**
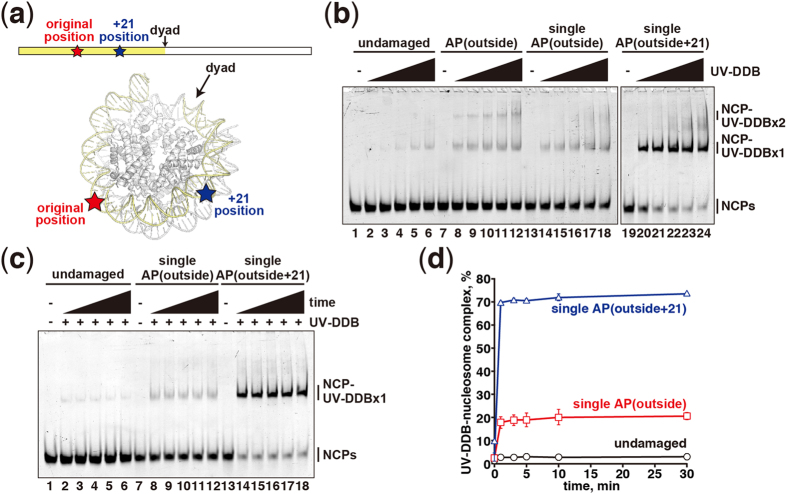
UV-DDB binds to the nucleosomal apyrimidinic DNA with a different translational position. (**a**) Schematic representations of reconstituted nucleosomes containing single AP(outside) and single AP(outside+21). The original AP(outside) position and the AP(outside+21) position are represented by red and blue stars, respectively. The arrow indicates the nucleosomal dyad. (**b**) Gel electrophoretic mobility shift assay for UV-DDB binding to single AP nucleosomes. Nucleosome core particles (NCP; 5 nM) containing undamaged DNA (lanes 1–6), AP(outside) (lanes 7–12), single AP(outside) (lanes 13–18), and AP(outside+21) (lanes 19—24) were incubated with UV-DDB. The UV-DDB concentrations are 0 nM (lanes 1, 7, and 13), 2.5 nM (lanes 2, 8, and 14), 5 nM (lanes 3, 9, and 15), 10 nM (lanes 4, 10, and 16), 20 nM (lanes 5, 11, and 17), and 40 nM (lanes 6, 12, and 18). (**c**) Time course experiments. Nucleosome core particles (NCP; 5 nM) containing undamaged DNA (lanes 1–6), single AP(outside) (lanes 7–12), and single AP(outside+21) (lanes 13–18) were incubated with UV-DDB (10 nM) for 1 min (lanes 2, 8, and 14), 3 min (lanes 3, 9, and 15), 5 min (lanes 4, 10, and 16), 10 min (lanes 5, 11, and 17), and 30 min (lanes 6, 12, and 18). Lanes 1, 7, and 13 are the experiments without UV-DDB. (**d**) Graphic representation of the time course experiments shown in panel (**c**). Standard deviation values are shown (n = 3).

We next tested the possibility that UV-DDB may bind to the AP regions partially unwrapped from the histone surface. To do so, we reconstituted the nucleosomes containing the AP lesion at a single site, located at either the original position (single AP(outside) nucleosome) or 21 base-pairs away from the original position, toward the nucleosomal dyad (single AP(outside+21) nucleosome) (Fig. 4a, Supplementary Figs S7 and S8). The upper band observed in the nucleosome containing two symmetric AP sites was absent in the analysis of the single AP(outside) nucleosome, indicating that the upper band corresponds to the nucleosome complexed with two UV-DDB molecules (Fig. 4b). To our surprise, we found that UV-DDB binding to the single AP(outside+21) nucleosome is robustly enhanced, as compared to that to the single AP(outside) nucleosome (Fig. 4b–d). The spontaneous unwrapping around the nucleosomal AP(outside+21) DNA region is reportedly extremely slow^18^, indicating that the UV-DDB binding occurs without unwrapping of the DNA from the histone core.

These results support the idea that the DNA flexibility at the damaged site of the nucleosome may provide the initial recognition parameter for UV-DDB. It should be noted that UV-DDB binding to the AP(outside) nucleosome was clearly weaker than that to the 6–4PP(outside) or 6–4PP(inside) nucleosome (Figs 1 and 3). These facts suggest that the existence of the flipped bases may stabilize the UV-DDB binding to damaged nucleosomes.

## Discussion

In the eukaryotic NER pathway, two damage surveillance protein complexes, UV-DDB and XPC, have been identified^2^,^19^,^20^,^21^,^22^,^23^. XPC recognizes a wide range of DNA lesions, including 6–4PP^24^,^25^. Nucleosome formation reportedly inhibits 6–4PP binding by XPC^26^ as well as excision of the lesion *in vitro*^27^,^28^, suggesting that the presence of a histone octamer may impede direct damage recognition by XPC and the following recruitment of other NER proteins. In contrast to XPC, we found that UV-DDB efficiently binds to nucleosomal 6–4PPs, even when located on a strand directly interacting with histones, probably by recognizing the DNA backbone flexibility at the damaged site.

While it is possible that the UV-DDB binding may occur at a damaged site that is partially unwrapped from the histone surface^18^,^29^, we consider this unlikely, because UV-DDB binding was enhanced when the damaged site was moved 21 base-pairs (+21) toward the nucleosomal dyad. This +21 position is stably wrapped around the histone octamer, and rarely unwraps spontaneously^18^. It is intriguing that UV-DDB exhibited better binding to the +21 position, as compared to the original position. According to the previous high-resolution crystal structure, the N-terminal H2A tail directly binds to the minor groove at the original position, but no histone tail interacts with the minor groove at the +21 position^30^ (Supplementary Fig. S9). Thus, this DNA-histone tail interaction may affect the UV-DDB binding to the nucleosomal damaged site.

UV-DDB binding to the flexible DNA region functions during the initial search for photolesions within chromatin. UV-DDB reportedly forms a stable complex with the flipped photodimer, which is accommodated within the specific binding pocket of DDB2^10^,^11^. After the initial coarse search process with the flexible DNA backbone, UV-DDB would adopt a more stable state by interacting with the flipped damaged bases, if appropriate damage is present. The nucleosome could be an obstacle to stable complex formation, and thus probably needs to be remodeled or removed.

UV-DDB associates *in vivo* with the CUL4 ubiquitin ligase^31^ and the CBP/p300 histone acetyltransferases^32^, suggesting that, after UV-DDB binds to the damaged site, the histone modifications catalyzed by these factors may promote the reorganization of the nucleosome structure. In response to UV irradiation, the CUL4 ubiquitin ligase reportedly ubiquitylates histones H3 and H4, which seemed to promote the dissociation of the modified histone octamer from the DNA^33^. The modification and removal of histones may then contribute to the conversion of the bound UV-DDB from the initial binding state to the stable binding state, followed by XPC recruitment.

In the XP-E patient cells lacking UV-DDB, the photolesions must be recognized in the absence of UV-DDB. Therefore, sliding and/or dissociation of histone octamers may be required for the efficient recognition of 6–4PPs by XPC, which could be facilitated by the intrinsic thermodynamic instability of the damaged nucleosomes^34^, with the aid of some chromatin remodeling factors. It is extremely intriguing to study the mechanism by which UV-DDB and XPC overcome the nucleosome barrier, after the initial UV-DDB binding to the damaged nucleosome, during the NER process.

## Methods

### Overexpression and purification of human histones

Human histones were expressed and purified as described previously^35^,^36^. The DNA fragment encoding the histone H2B T122C mutant, in which the Thr122 residue was replaced by Cys, was constructed by site-directed mutagenesis, and the H2B T122C mutant was prepared by the method described previously^35^,^36^. Reconstitution and purification of the H2A-H2B T122C complex, the H3.1-H4 complex, and the histone octamer were performed as described previously^35^,^36^,^37^. Freeze-dried histones were mixed at an equal molar ratio in 20 mM Tris-HCl (pH 7.5) buffer, containing 7 M guanidine hydrochloride and 20 mM 2-mercaptoethanol. Samples were dialyzed against 10 mM Tris-HCl (pH 7.5) buffer, containing 2 M NaCl, 1 mM EDTA, and 2 mM 2-mercaptoethanol. The resulting histone complexes were purified by Superdex 200 gel filtration chromatography.

### Fluorescent labeling of the H2A-H2B complex

The purified H2A-H2B T122C complex (46 μM) was conjugated with a fluorescent dye, using 558 μM of Alexa Fluor 488 C_5_ Maleimide (Invitrogen) in 10 mM Tris-HCl (pH 7.5) buffer, containing 2 M NaCl, 1 mM EDTA, and 1 mM TCEP, at room temperature for 2 h. The dried fluorescent dye (1 mg) was dissolved in 200 μl of DMSO. The reaction was stopped by the addition of 159 mM 2-mercaptoethanol, and the sample was then dialyzed against 10 mM Tris-HCl (pH 7.5) buffer, containing 2 M NaCl, 1 mM EDTA, and 5 mM 2-mercaptoethanol.

### Preparation of DNAs

The oligonucleotides containing the 6–4PP (6–4PP ssDNA) were synthesized with the 6–4PP building block, using an Applied Biosystems 3400 DNA synthesizer^38^. Benzimidazolium triflate was used as an activator on Universal Support II PS (Glen Research), as described previously^39^. For the preparation of the 5′-phosphorylated oligonucleotide, Chemical Phosphorylation Reagent (Glen Research) was used on the synthesizer, and at the deprotection step, the treatment with ammonia water at room temperature was prolonged to 6 h to remove the protecting group for the 5′-phosphate. After deprotection, the products were purified by HPLC using a Waters μBondasphere C18 15 μm 300A column (7.8 × 300 mm) at 60 °C, with a linear gradient of acetonitrile in 0.1 M triethylammonium acetate (pH 7.0). The eluate was concentrated *in vacuo*, and after desalting on an illustra NAP-10 column (GE Healthcare), the counter cation was exchanged to Na^+^ using AG 50W-X2 resin (Bio-Rad).

The single-stranded DNA containing the d-Spacer/THF (AP ssDNA) and the complementary single-stranded DNA without DNA damage (complementary ssDNA) were purchased from Tsukuba Oligo Service or FASMAC, Japan. The complementary ssDNA was mixed with the 6–4PP(inside) ssDNA, 6–4PP(outside) ssDNA, AP(inside) ssDNA, AP(outside) ssDNA, or AP(outside+21) ssDNA in a 1:1 ratio, and the double-stranded DNA (dsDNA) was formed by annealing. The 6–4PP(outside) dsDNA contained a four-base overhang, 5′-AATT-3′, at one end for self-ligation. The 6–4PP(inside), AP(inside), and AP(outside) dsDNAs contained a three-base overhang, 5′-GTT-3′ or 5′-AAC-3′, at one end for ligation. For the single AP(outside) and single AP(outside+21) dsDNAs, a lesion was introduced into the dsDNA containing a three-base overhang, 5′-AAC-3′. The resulting 146-mer dsDNA contains the α-satellite DNA sequence used in the previous X-ray analyses of the nucleosome structures^13^,^35^. The resulting 145-mer dsDNA contains the same nucleotide sequence as the 146-mer dsDNA, except for a three-base overhang site for ligation and damaged sites. The 146-mer 6–4PP(outside), 145-mer 6–4PP(outside), 145-mer AP(inside), 145-mer AP(outside), 145-mer single AP(outside), and 145-mer single AP(outside+21) dsDNAs were then prepared by ligation. The resulting dsDNAs contained the 6–4PP or apyrimidinic lesions at either symmetric positions or asymmetric single positions (Supplementary Figs S1 and S7).

### Preparation of nucleosomes

For the nucleosomes containing the fluorescently labeled histone H2B, dsDNA, the H3.1-H4 complex, and the Alexa488 labeled H2B T122C-H2A complex were mixed in a 1:4:5.4 molar ratio in the presence of 2 M KCl. For the nucleosomes without the fluorescently labeled histone H2B, the dsDNA and a histone octamer, containing H2A, H2B, H3.1, and H4, were mixed in a 1:1.9 molar ratio in the presence of 2 M KCl. The samples were then dialyzed against 10 mM Tris-HCl (pH 7.5) buffer, containing 2 M KCl, 1 mM EDTA, and 1 mM DTT. The KCl concentration was gradually reduced from 2 M to 0.25 M using a peristaltic pump with 10 mM Tris-HCl (pH 7.5) buffer, containing 0.25 M KCl, 1 mM EDTA, and 1 mM DTT, at 4 °C. Samples were further dialyzed against 10 mM Tris-HCl (pH 7.5) buffer, containing 0.25 M KCl, 1 mM EDTA, and 1 mM DTT, at 4 °C for 4 h. The reconstituted nucleosomes were incubated at 55 °C for 2 h, and the non-specific complexes formed between free DNA and histones were removed. After the 55 °C treatment, the nucleosomes were purified by native polyacrylamide gel electrophoresis using a Prep Cell apparatus (Bio-Rad). For crystallization of the nucleosomes, the dsDNA and a histone octamer were mixed in a 1:1.1 molar ratio for the 6–4PP(inside) nucleosome and in a 1:1.3 molar ratio for the 6–4PP(outside) nucleosome, in the presence of 2 M KCl. The nucleosomes were reconstituted and purified as described above, and then the purified nucleosomes were dialyzed against 20 mM potassium cacodylate buffer (pH 6.0), containing 1 mM EDTA.

### Purification of the human UV-DDB protein

Recombinant human UV-DDB (DDB1-DDB2 heterodimer) was expressed in insect cells, and was purified as described previously^23^.

### Gel electrophoretic mobility shift assay

For the DNA binding assay, DNA (20 nM) was mixed with UV-DDB (0, 5, 10, 20, 30, and 40 nM) in the presence of 91.4 pM of ϕX174 supercoiled DNA (New England BioLabs). The reactions were conducted in 28 mM sodium phosphate (pH 7.5), containing 150 mM NaCl, 3.4 mM MgCl_2_, 1.4 mM EDTA, 2% glycerol, 0.014% Triton X-100, 0.1 mg/ml BSA, and 1 mM DTT, at 30 °C for 30 min. For the nucleosome binding assay, each nucleosome (5 nM) containing a fluorescently labeled histone was mixed with UV-DDB (0, 2.5, 5, 10, 20, and 40 nM), in the presence of 91.4 pM of ϕX174 supercoiled DNA (New England BioLabs) and 19-fold excess unlabeled nucleosome without DNA lesions. The reactions were conducted in 28 mM sodium phosphate (pH 7.5), containing 150 mM NaCl, 3.4 mM MgCl_2_, 1.4 mM EDTA, 2% glycerol, 0.014% Triton X-100, 0.1 mg/ml BSA, and 1 mM DTT, and incubated at 30 °C for 30 min. For time course experiments, the reactions were incubated at 30 °C for 1, 5, 10, and 30 min in the presence of UV-DDB (10 nM). After the incubation, the samples were analyzed by electrophoresis on a 6% non-denaturing polyacrylamide gel (acrylamide:bis = 37.5:1) in 0.5 × TGE buffer (12.5 mM Tris base, 96 mM glycine, and 0.5 mM EDTA), and the bands were visualized with a Typhoon 9410 imaging analyzer (GE Healthcare).

### Crystallization and structure determination

Crystals of the purified 6–4PP(inside) and 6–4PP(outside) nucleosomes were obtained by the hanging drop method, after mixing equal volumes of the nucleosome solution and 20 mM potassium cacodylate buffer (pH 6.0), containing 50 mM KCl and 85–120 mM MnCl_2_. The 6–4PP(inside) and 6–4PP(outside) nucleosome samples were equilibrated against a reservoir solution, containing 20 mM potassium cacodylate (pH 6.0), 35–45 mM KCl, and 55–65 mM MnCl_2_. The crystals of the 6–4PP(inside) and 6–4PP(outside) nucleosomes were soaked in a cryo-protectant solution, containing 20 mM potassium cacodylate (pH 6.0), 40 mM KCl, 55–65 mM MnCl_2_, 30% (+/−)-2-methyl–2,4-pentanediol, and 2% trehalose. The crystals were flash-cooled in a stream of N_2_ gas (100 K). The 6–4PP(inside) and 6–4PP(outside) nucleosome crystals belonged to the orthorhombic space group *P*2_1_2_1_2_1_, and contained one nucleosome per asymmetric unit. Diffraction data were collected using the synchrotron radiation source at the beamline BL41XU station of SPring-8 and the BL-17A station of the Photon Factory.

The diffraction data of the 6–4PP(inside) and 6–4PP(outside) nucleosomes were integrated and scaled with the HKL2000 program^40^. The data were processed with the CCP4 program suite^41^. The structures were solved by the molecular replacement method, using the Phaser program^42^ with the human nucleosome structure (PDB ID: 3AFA) as the search model. The structures of the 6–4PP(inside) and 6–4PP(outside) nucleosomes were initially calculated at 4.0 Å and 3.5 Å resolutions, respectively. Rigid body refinement of the obtained solution was performed using the Phenix program^43^. Further structural refinement consisted of iterative rounds of energy minimization and B factor refinement using the Phenix program^43^, and model building using the COOT program^44^. The Ramachandran plot of the final 6–4PP(inside) nucleosome structure showed 100% of the residues in the most favorable and additional allowed regions, and no residues in the disallowed region. Similarly, the Ramachandran plot of the final 6–4PP(outside) nucleosome structure showed 99.4% of the residues in the most favorable and additional allowed regions, and no residues in the disallowed region. Summaries of the data collection and refinement statistics are provided in Supplementary Table S1. All structure figures were created using the PyMOL program (http://pymol.org). The atomic coordinates of the 6–4PP(inside) and 6–4PP(outside) nucleosomes have been deposited in the RCSB, with the ID codes 4YM5 and 4YM6, respectively.

## Additional Information

**Accession Codes:** The atomic coordinates of the 6-4PP(inside) and 6-4PP(outside) nucleosomes have been deposited in the Protein Data Bank, under the accession codes 4YM5 and 4YM6, respectively.

**How to cite this article**: Osakabe, A. *et al.* Structural basis of pyrimidine-pyrimidone (6–4) photoproduct recognition by UV-DDB in the nucleosome. *Sci. Rep.* **5**, 16330; doi: 10.1038/srep16330 (2015).

## Supplementary Material

Supplementary Information
