# Supplementary material for: Structural basis of pyrimidine-pyrimidone (6–4) photoproduct recognition by UV-DDB in the nucleosome
Source: Sci Rep. 2015 Nov 17;5:16330. doi: 10.1038/srep16330 (PMC4648065; doi:10.1038/srep16330)
Supplement: Supplementary Information [file srep16330-s1.pdf]

Supplementary Information

Structural basis of pyrimidine-pyrimidone (6-4) photoproduct recognition by UV-DDB in the nucleosome

Akihisa Osakabe, Hiroaki Tachiwana, Wataru Kagawa, Naoki Horikoshi, Syota Matsumoto, Mayu Hasegawa, Naoyuki Matsumoto, Tatsuya Toga, Junpei Yamamoto, Fumio Hanaoka, Nicolas H. Thomä, Kaoru Sugasawa, Shigenori Iwai, and Hitoshi Kurumizaka

Supplementary Figures S1-S9

Supplementary Table S1

Supplementary Figures and Legends

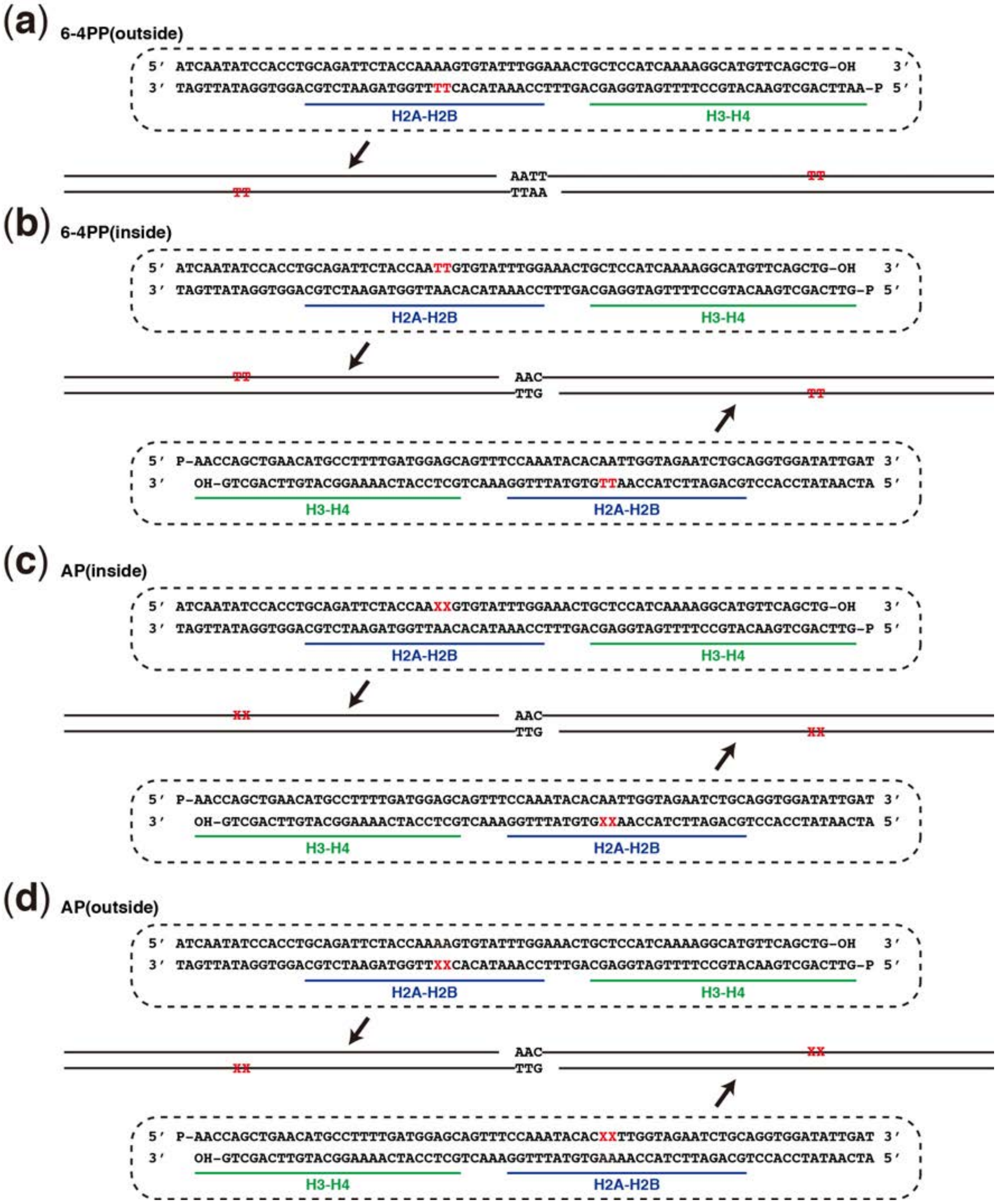

Supplementary Figure S1. The 6-4PP and AP DNA substrates used for nucleosome reconstitution.

(**a**) 6-4PP(outside) DNA. The 71-mer ssDNA was annealed to the complementary 75-mer ssDNA, which contains the 6-4PP bases at positions 46 and 47 from the 5’ end of the strand. The affected T-T bases are colored red. Green and blue lines indicate the regions that interact with H3-H4 and H2A-

H2B, respectively. The resulting dsDNA contained a 5'-AATT-3' overhang with a phosphate at the 5' end. The 146 base-pair dsDNA was prepared by self-ligation of the dsDNA 71-mer containing the 5'-AATT-3' overhang. (**b**) 6-4PP(inside) DNA. The 74-mer ssDNAs were annealed to the complementary 71-mer ssDNA, which contains the 6-4PP bases at positions 29 and 30 from the 5' end of the strand. The affected T-T bases are colored red. The resulting dsDNAs contained 5'-AAC-3' and 5'-GTT-3' overhangs with a phosphate at the 5' end. The 145 base-pair dsDNA was prepared by ligation of these dsDNA 71-mers containing the 5'-AAC-3' and 5'-GTT-3' overhangs. (**c**) AP(inside) DNA. The 74-mer ssDNA was annealed to the complementary 71-mer ssDNA, which contains the apyrimidinic region at positions 29 and 30 from the 5' end of the strand. The apyrimidinic sites are indicated by red Xs. The resulting dsDNAs contained 5'-AAC-3' and 5'-GTT-3' overhangs with a phosphate at the 5' end. The 145 base-pair dsDNA was prepared by ligation of these dsDNA 71-mers containing the 5'-AAC-3' and 5'-GTT-3' overhangs. (**d**) AP(outside) DNA. The 71-mer ssDNA was annealed to the complementary 74-mer ssDNA, which contains the apyrimidinic region at positions 45 and 46 from the 5' end of the strand. The apyrimidinic sites are indicated by red Xs. The resulting dsDNAs contained 5'-AAC-3' and 5'-GTT-3' overhangs with a phosphate at the 5' end. The 145 base-pair dsDNA was prepared by ligation of these dsDNA 71-mers containing the 5'-AAC-3' and 5'-GTT-3' overhangs.

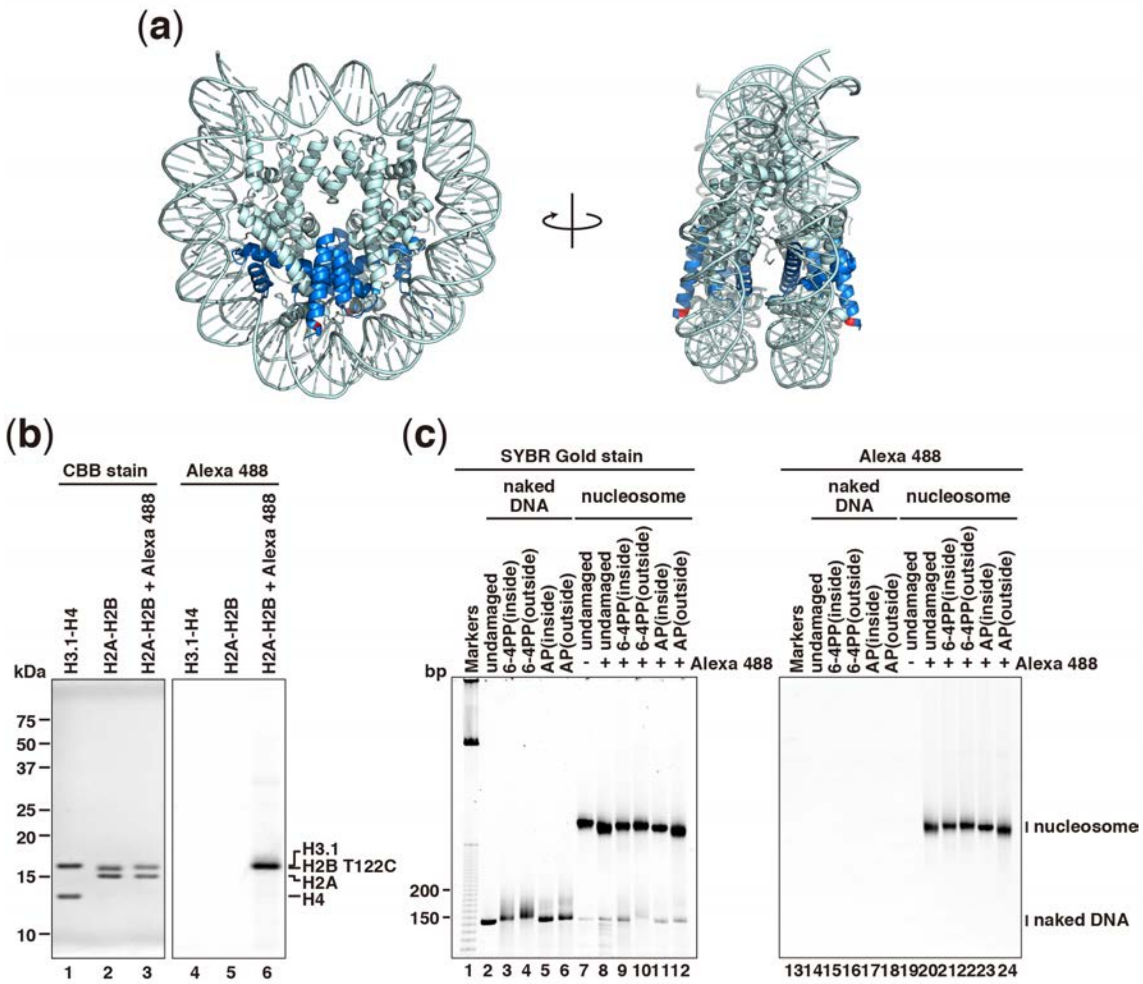

Supplementary Figure S2. Preparation of nucleosomes containing fluorescently labeled histone H2B.

(**a**) Locations of the H2B Thr122 residue labeled by Alexa Fluor 488 fluorescent dye in the nucleosome structure (PDB ID: 3AFA). Two views of the nucleosome are presented, and the H2B Thr122 residues are colored red. (**b**) Purified histone complexes were analyzed by 16% SDS-PAGE with Coomassie Brilliant Blue staining (lanes 1-3) and with the Alexa488 signal (lanes 4-6) detected with a Typhoon 9410 imager (GE Healthcare). Lanes 1 and 4 indicate the H3-H4 complex. Lanes 2 and 5 indicate the H2A-H2B T122C complex without Alexa488. Lanes 3 and 6 indicate the H2A-Alexa488 labeled H2B T122C complex. (**c**) Purified nucleosomes were analyzed by non-denaturing 6% PAGE with SYBR Gold (Invitrogen) staining (lanes 1-12) and with the Alexa488 signal (lanes 13-24) detected with a Typhoon 9410 imager (GE Healthcare). Lanes 1 and 13 indicate 10 base-pair DNA ladder markers. Lanes 2 and 14 indicate

undamaged DNA. Lanes 3 and 15 indicate 6-4PP(inside) DNA. Lanes 4 and 16 indicate 6-4PP(outside) DNA. Lanes 5 and 17 indicate AP(inside) DNA. Lanes 6 and 18 indicate AP(outside) DNA. Lanes 7 and 19 indicate undamaged nucleosomes without the fluorescently labeled histone. Lanes 8-12 and 20-24 indicate nucleosomes containing the Alexa488-labeled H2B. Lanes 8 and 20 indicate undamaged nucleosomes. Lanes 9 and 21 indicate 6-4PP(inside) nucleosomes. Lanes 10 and 22 indicate 6-4PP(outside) nucleosomes. Lanes 11 and 23 indicate AP(inside) nucleosomes. Lanes 12 and 24 indicate AP(outside) nucleosomes.

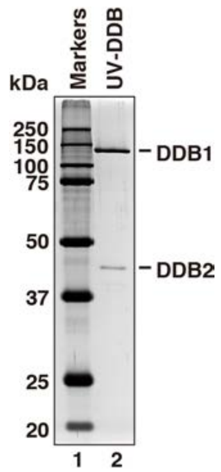

Supplementary Figure S3. SDS-PAGE analysis of purified UV-DDB.
Purified UV-DDB (DDB1-DDB2) was analyzed by 12% SDS-PAGE with silver staining. Lanes 1 and 2 indicate the molecular mass markers and the purified UV-DDB, respectively.

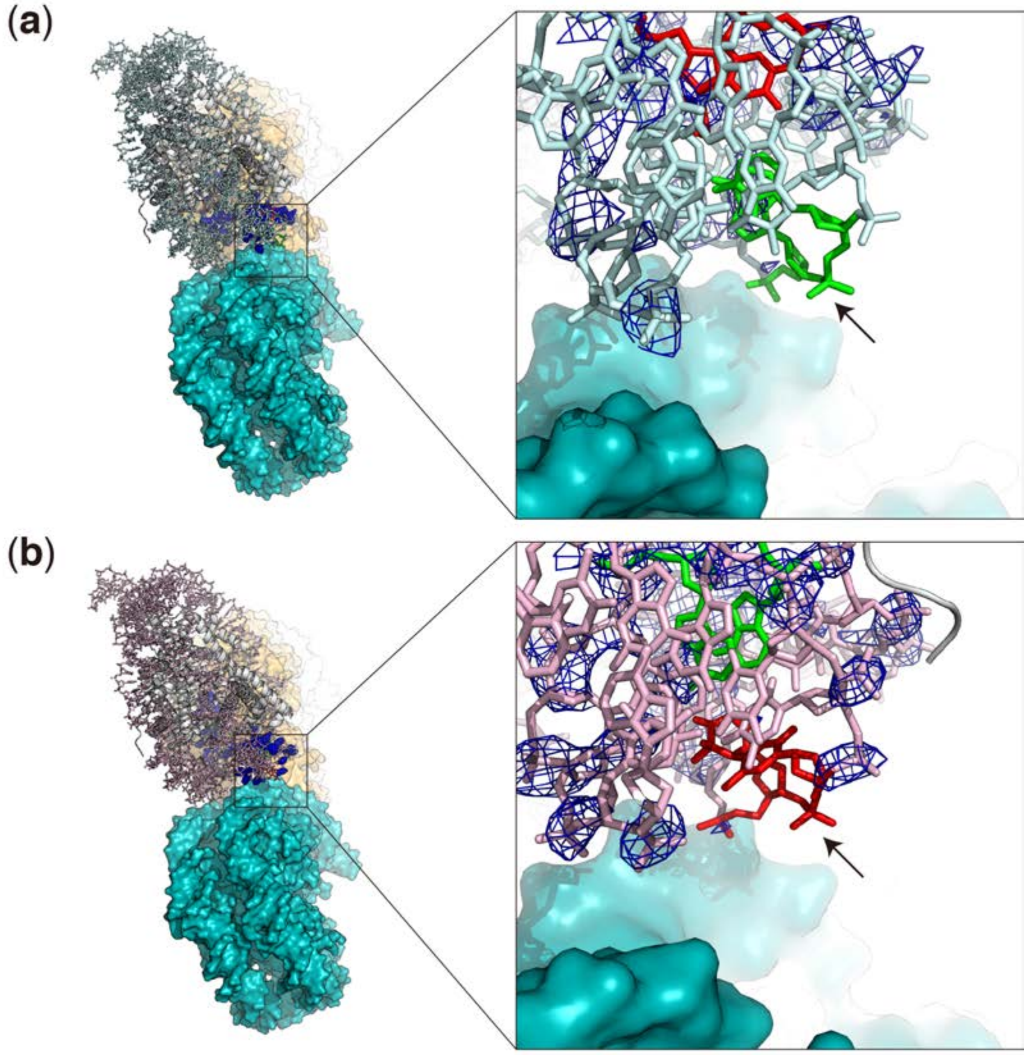

Supplementary Figure S4. Crystal packing environment of the 6-4PP sites within the nucleosome.

(**a**) Views of the 6-4PP(inside) site in the nucleosome structure. The arrow indicates the complementary strand (green) of the 6-4PP lesion (red). (**b**) Views of the 6-4PP(outside) site in the nucleosome structure. The arrow indicates the 6-4PP lesion (red). The electron densities of the 4 bases (contoured at 1.5 σ) around each affected site are shown in blue. The neighboring nucleosome in the crystal is shown by a space filling representation, colored bright blue and bright orange.

(a) (b) (c)

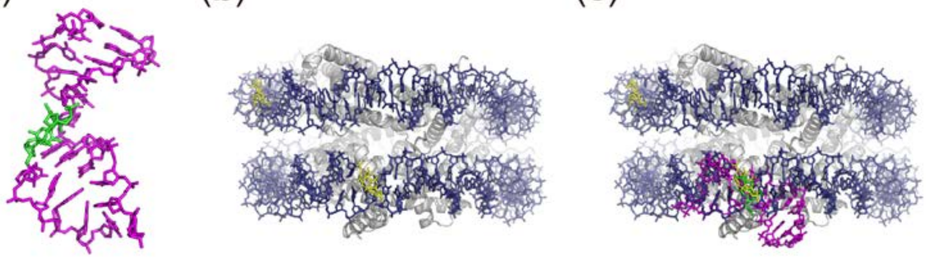

Supplementary Figure S5. Superimposition of the kinked 6-4PP DNA with the nucleosome containing 6-4PP(outside).
(**a**) The kinked 6-4PP DNA structure, modeled according to the previous NMR structure published by Kim and Choi (1995). The 6-4PP bases are colored green. (**b**) Side view of the 6-4PP(outside) nucleosome. The affected T-T bases are colored yellow. (**c**) The modeled 6-4PP DNA shown in panel (**a**) is superimposed on the 6-4PP(outside) nucleosome (**b**).

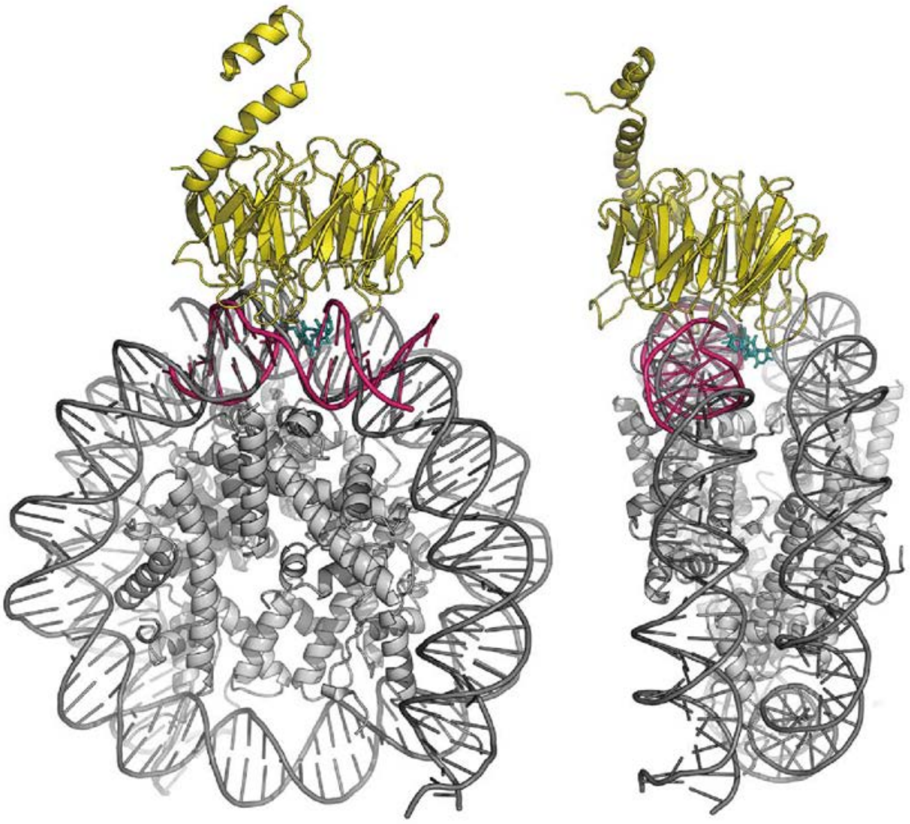

Supplementary Figure S6. Model of the UV-DDB and 6-4PP nucleosome complex.

The structure of the DNA-binding subunit of UV-DDB, DDB2, complexed with the 6-4PP DNA is superimposed on the disordered 6-4PP(outside) site of the nucleosome structure. The DDB2 and the 6-4PP DNA in the complex are colored yellow and magenta, respectively. The affected 6-4PP residues are colored light blue.

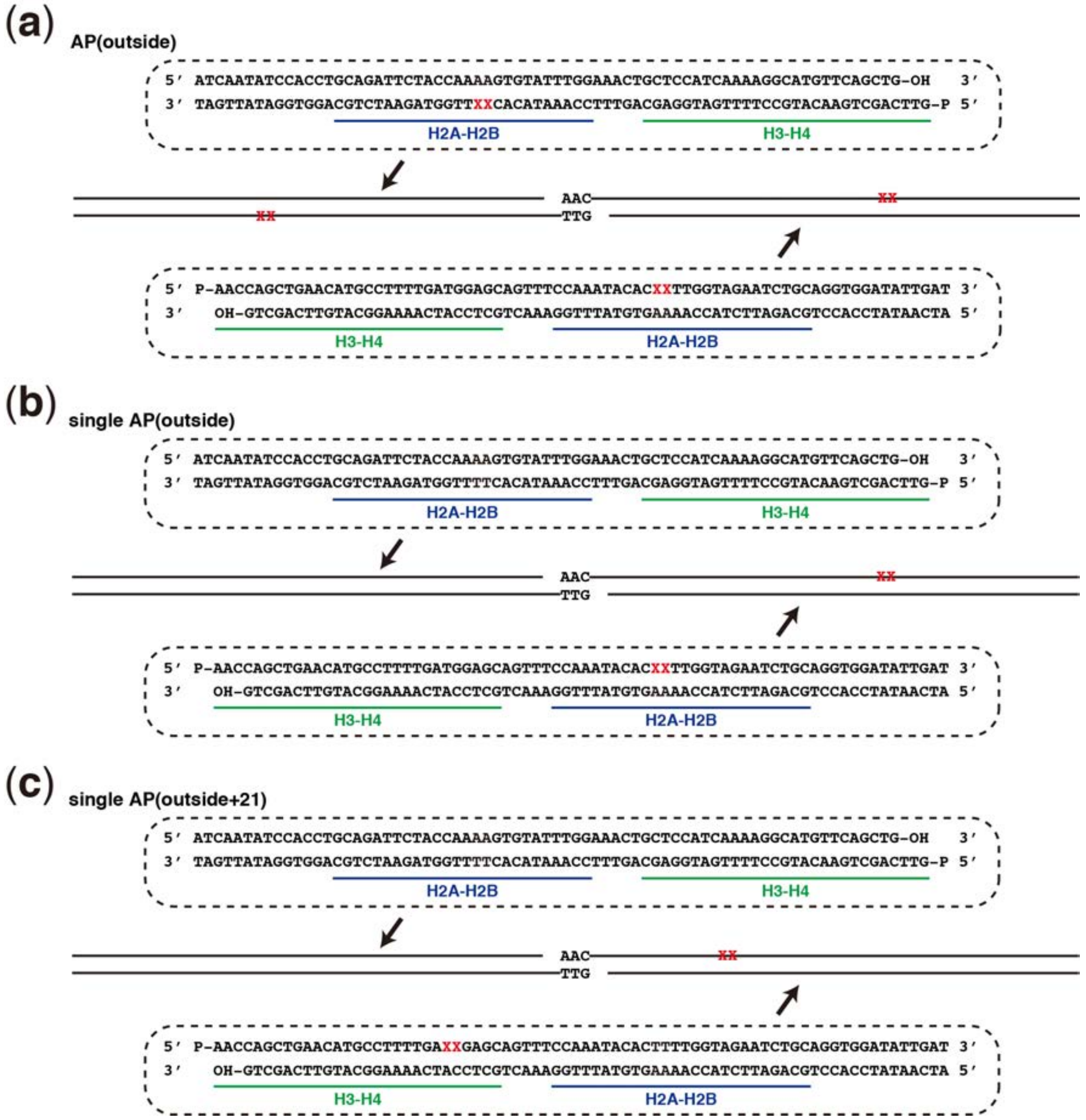

Supplementary Figure S7. The single AP DNA substrates used for nucleosome reconstitution.

(**a**) AP(outside) DNA. The 71-mer ssDNA was annealed to the complementary 74-mer ssDNA, which contains the apyrimidinic region at positions 45 and 46 from the 5’ end of the strand. The apyrimidinic sites are indicated by red Xs. The resulting dsDNAs contained 5’-AAC-3’ and 5’-GTT-3’ overhangs with a phosphate at the 5’ end. The 145 base-pair dsDNA was prepared by ligation of these dsDNA 71-mers containing the 5’-AAC-3’ and 5’-GTT-3’ overhangs. Green and blue lines indicate the regions that interact with H3-H4 and H2A-H2B, respectively. (**b**) Single AP(outside) DNA. The apyrimidinic sites are

indicated by red Xs. The apyrimidinic bases were introduced into a 74-mer ssDNA containing a 5'-AAC-3' overhang at positions 45 and 46 from the 5' end of the strand. The resulting dsDNAs contained 5'-AAC-3' and 5'-GTT-3' overhangs, with a phosphate at the 5' end. The 145 base-pair dsDNA was prepared by ligation of these dsDNA 71-mers, containing the 5'-AAC-3' and 5'-GTT-3' overhangs. (**c**) Single AP(outside+21) DNA. The apyrimidinic sites are indicated by red Xs. The apyrimidinic bases were introduced into a 74-mer ssDNA containing a 5'-AAC-3' overhang at positions 24 and 25 from the 5' end of the strand. The resulting dsDNAs contained 5'-AAC-3' and 5'-GTT-3' overhangs, with a phosphate at the 5' end. The 145 base-pair dsDNA was prepared by ligation of these dsDNA 71-mers, containing the 5'-AAC-3' and 5'-GTT-3' overhangs.

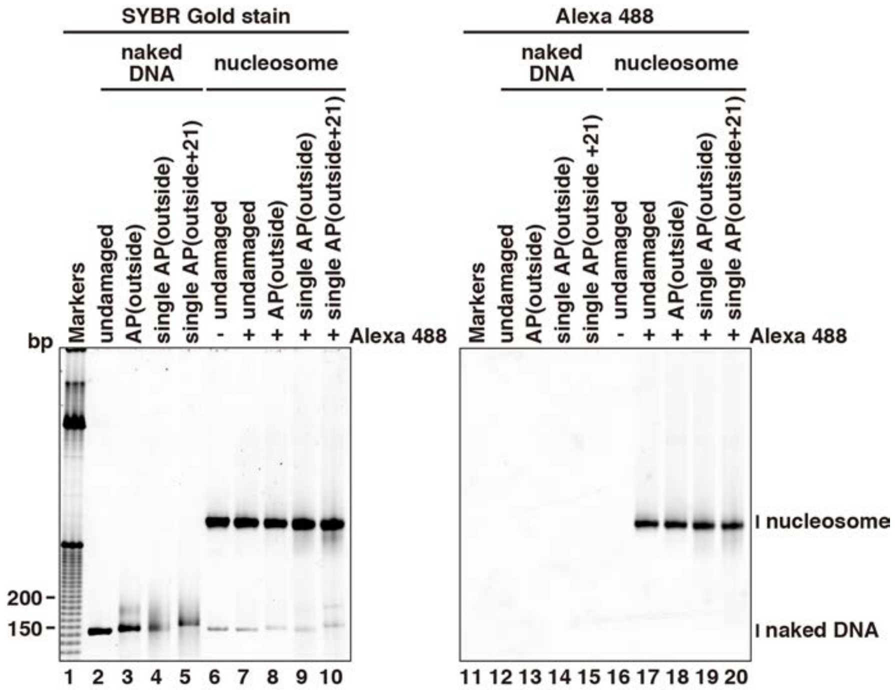

Supplementary Figure S8. Preparation of nucleosomes containing single AP(outside) with fluorescently labeled histone H2B.

(**a**) Purified nucleosomes were analyzed by non-denaturing 6% PAGE with SYBR Gold (Invitrogen) staining (lanes 1-10) and with the Alexa488 signal (lanes 11-20) detected with a Typhoon 9410 imager (GE Healthcare). Lanes 1 and 11 indicate 10 base-pair DNA ladder markers. Lanes 2 and 12 indicate undamaged DNA. Lanes 3 and 13 indicate AP(outside) DNA. Lanes 4 and 14 indicate single AP(outside) DNA. Lanes 5 and 15 indicate single AP(outside+21) DNA. Lanes 6 and 16 indicate undamaged nucleosomes without the fluorescently labeled histone. Lanes 7-10 and 17-20 indicate nucleosomes containing the Alexa488-labeled H2B. Lanes 7 and 17 indicate undamaged nucleosomes. Lanes 8 and 18 indicate AP(outside) nucleosomes. Lanes 9 and 19 indicate single AP(outside) nucleosomes. Lanes 10 and 20 indicate single AP(outside+21) nucleosomes.

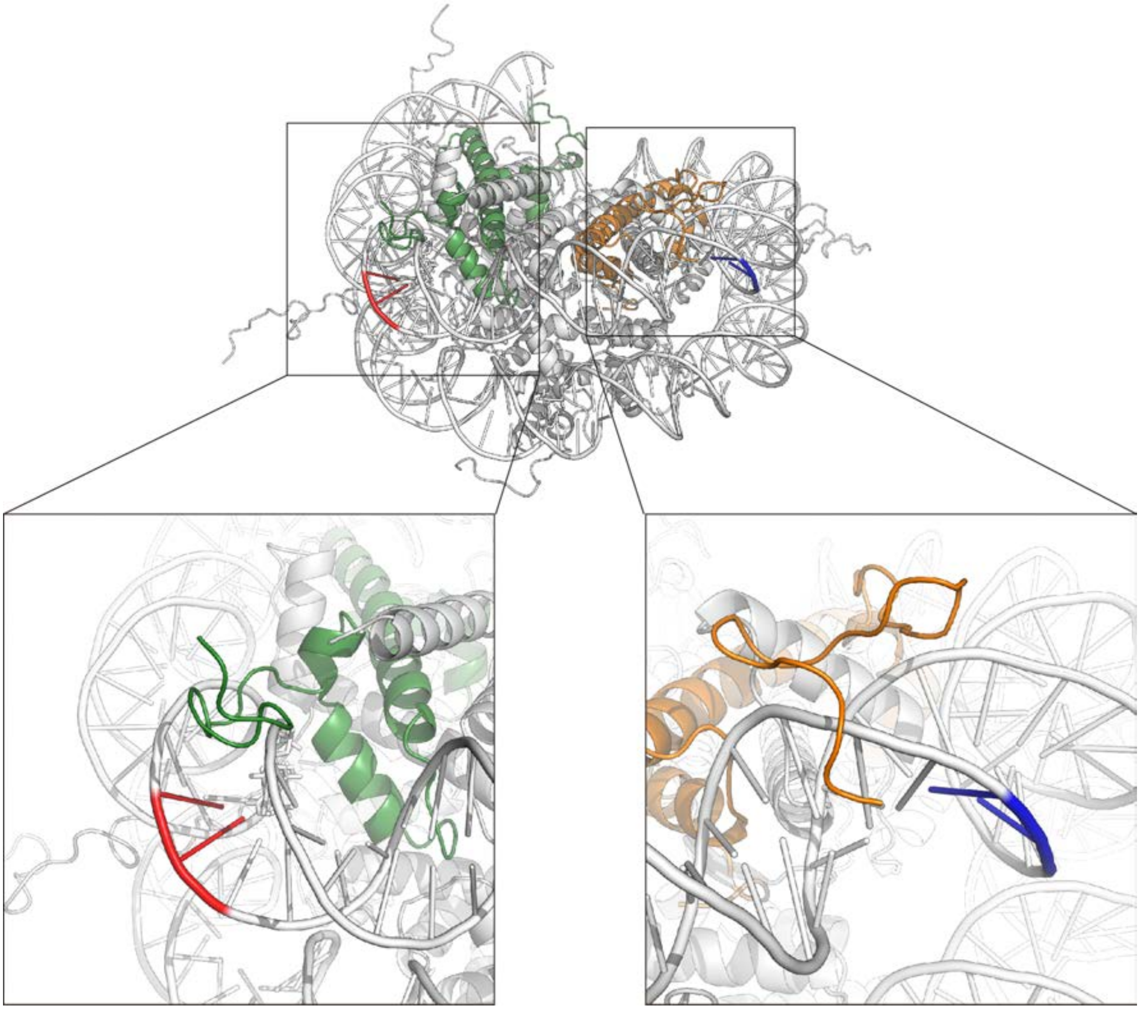

Supplementary Figure S9. Locations of apyrimidinic sites superimposed on the high resolution crystal structure of the nucleosome (PDB ID:1kx5). Two close-up views of the original AP(outside) position (red) and the AP(outside+21) position (blue). H2A and H4 are colored green and orange, respectively. The N-terminal tail of H2A binds the minor groove of the original AP(outside) region (left panel).

Supplementary Table S1 Data collection and refinement statistics (**Molecular replacement**)

	6-4PP(outside)	6-4PP(inside)
**Data collection**		
Space group	$P2_12_12_1$	$P2_12_12_1$
Cell dimensions		
a, b, c (Å)	105.7, 109.5, 178.2	104.2, 109.4, 174.4
a, b, g (°)	90, 90, 90	90, 90, 90
Resolution (Å) *	50.0-3.5 (3.63-3.50)	50.0-4.0 (4.14-4.00)
No. of unique reflections	26,130	17,241
R_{sym} or R_{merge} *, **	9.6 (55.1)	8.8 (45.3)
I/sI *	9.3 (4.2)	9.1 (5.1)
Completeness (%) *	99.6 (100)	99.6 (100)
Redundancy *	6.7 (6.9)	6.2 (6.5)
**Refinement**		
Resolution (Å)	29.8-3.5	33.2-4.0
No. reflections	25,295	17,161
R_{work}/R_{free} ***	24.4/28.7	20.3/28.9
No. atoms		
Protein	6,027	6,039
Ligand/ion	5,980	5,939
Water	-	-
B-factors		
Protein	104.1	131.5
Ligand/ion	199.6	213.4
Water	-	-
R.m.s. deviations		
Bond lengths	0.019	0.011
Bond angles	1.34	1.35
PDB ID	4YM6	4YM5

*Highest resolution shell is shown in parentheses.
**Rsym = ΣhklΣI IIhkl,i – <Ihkl>I / ΣhklΣI Ihkl,i.
***Rwork = ΣhklIIFoI-IFcII / ΣhklIFoI.
Rfree was calculated with 5% of the data excluded from the refinement.
